# Associating Renewable Energy, Globalization, Agriculture, and Ecological Footprints: Implications for Sustainable Environment in South Asian Countries

**DOI:** 10.3390/ijerph191610162

**Published:** 2022-08-16

**Authors:** Lixun Wang, Usman Mehmood, Ephraim Bonah Agyekum, Solomon Eghosa Uhunamure, Karabo Shale

**Affiliations:** 1 Terms in Financial Engineering School of Economics and Management, Weifang University of Science and Technology, Weifang 262799, China; 2 Department of Political Science, University of Management and Technology, Lahore 54590, Pakistan; 3 Remote Sensing, GIS and Climatic Research Laboratory (National Center of GIS and Space Applications), Centre for Remote Sensing, University of the Punjab, Lahore 54590, Pakistan; 4 Department of Nuclear and Renewable Energy, Ural Federal University Named after the First President of Russia Boris Yeltsin, 19 Mira Street, 620002 Ekaterinburg, Russia; 5 Faculty of Applied Sciences, Cape Peninsula University of Technology, P.O. Box 652, Cape Town 8000, South Africa

**Keywords:** ecological footprint, CO_2_ emissions, renewable energy, globalization, South Asian countries

## Abstract

The main purpose of this work is to investigate the impacts of globalization (GL), renewable energy (RE), and value-added agriculture (AG) on ecological footprints (EF) and CO_2_ emissions. For quantitative analysis, this research paper includes yearly data from 1990–2018 for four South Asian nations: Bangladesh, India, Pakistan, and Sri Lanka. These countries are most vulnerable to climate hazards and rapid economic transitions. The Westerlund test provides a strong association among the panel data. The findings of ordinary least squares (DOLS) and fully modified ordinary least squares (FMOLS) show that RE is lowering CO_2_ emissions and EF in the long run. A 1% increase in RE results in a 10.55% and 2.08% CO_2_ decrease in emissions and EF, respectively. Globalization and AG are contributing to environmental degradation in selected South Asian countries. Therefore, these countries need to exploit solar energy to its full capacity. Moreover, these countries need to explore more RE resources to reduce their dependence on non-RE sources. These countries can make their agricultural sectors sustainable by following efficient farming practices. Environmental awareness should be enhanced among the farmers. Farmers can use animal fertilizers and clean inputs in AG to achieve sustainable agricultural products. Overall, this work suggests that these countries can achieve a cleaner environment by adopting RE and by promoting efficient technologies through globalization.

## 1. Introduction

Over the last few years, the world has seen a rapid increase in global temperature [1,2,3]. Carbon dioxide (CO_2_) emissions make up the largest share of greenhouse gasses (GHG). The concentration of CO_2_ emissions has increased by 146% in the last few years [4,5,6]. Along with CO_2_ emissions, there are other polluting factors which are creating environmental problems. In this context, the authors of [7] presented the term EF as an accumulative environmental factor that considers water, air, and soil pollution. EF considers the human pressures on the environment according to six parameters regarding grazing land, cropland, fishing, and carbon grounds. Ever since the 1970s, EF has been increasing according to the biocapacity of the world, which is now creating drastic climatic problems, especially in developing nations. This index calculates the human necessity for environmental resources through the use of ecosystem services rooted in the earth. Explicitly, this index is identified as a physical index rather than a financial quantity because it measures the use of naturally beneficial land to meet human demands and to deal with human-generated waste. According to the measurement from www.footprintnetwork.org, this index is quantified in units of global hectares area (gha). Therefore, this index measures the usage of environmental services through human activities such as construction, agriculture, raising livestock, and fishing.

**Figure 1:**
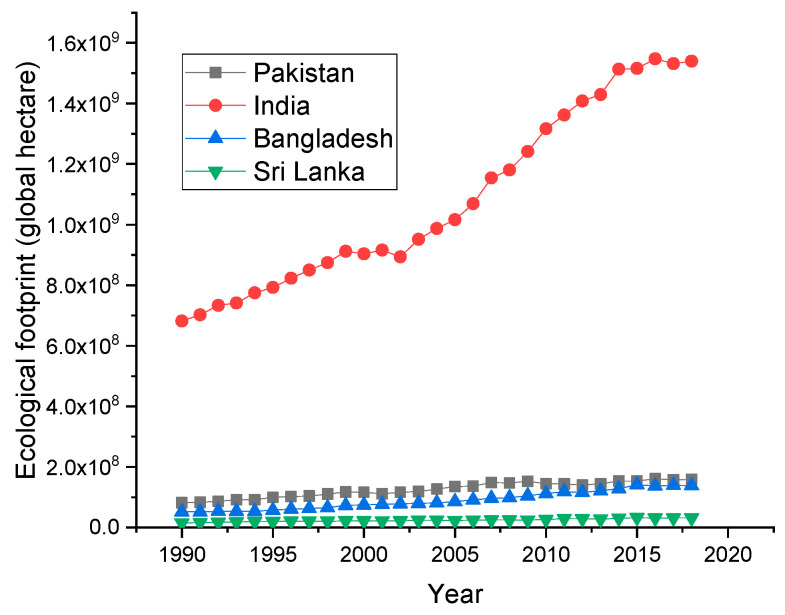
Trends of ecological footprints (Source: Global Footprint Network).

Figure 1 shows the trend of EF in selected South Asian nations. Sri Lanka’s EF is comparatively lower than the other countries. The reason may be that Sri Lanka is depending upon more imports than exports. The lower level of exports may be due to the lower production of domestic products. The other countries are exporting their domestic goods rapidly. Therefore, they have huge industries, and their ecological footprints are comparatively high.

Today, the world is trying to achieve green growth, which means fulfilling the needs of the nations without hurting the ecosystem [8]. According to the Brundtland report in 1987, increasing population, energy use, and excessive use of natural resources in agricultural and industrial production are the main hurdles in the way of sustainable development [9].

AG is considered an important tool to achieve sustainable development [10]. The world has acknowledged the role of AG to eradicate poverty. Therefore, it is important to produce sustainable food with an efficient supply of nutritional products. In this context, an efficient transformation of the agricultural system, which considers environmental protection, is required today.

Agricultural activities support economies by providing food and jobs to people. Agricultural activities, such as forestry and fishing, play a significant role in wealth creation, especially in developing countries [11,12]. However, these activities increase water consumption and carbon footprints. Due to the food production of maize, wheat, rice, and livestock, carbon footprints have increased over the past few decades [13,14,15]. Agricultural production using traditional technologies is causing environmental problems, which are becoming hurdles in the way of sustainable development goals [13,16]. Therefore, it is important to reduce carbon emissions, which are emitted from agricultural outputs. AG is the third contributing factor to environmental pollution, after energy and industry [17]. Activities such as crop and soil burning and the usage of fossil fuels are contaminating the environment [18].

During the COVID-19 pandemic, the energy requirements were significantly reduced, and the world’s economies saw environmental improvements. According to the research of [19], the COVID-19 situation reduced the pace of economic growth and reduced fossil fuel consumption. This situation further improves environmental quality. The scholars recommended the use of RE as an effective tool to mitigate environmental pollution. Therefore, fossil fuels are contaminating the climate, and RE provides the advantage to the farmers to compensate for the economic and climatic losses [18,20]. RE can be used for different purposes, such as heating, cooling, and irrigation systems [21]. Today, developed and developing nations are experiencing rapid population growth. Due to better health and employment opportunities, people are moving toward urban areas. Urban areas require huge infrastructural settlements, which consume more natural resources. If these settlements are created without planning and the energy is consumed without regard to renewable resources, the environmental quality will degrade significantly. Therefore, rapid population growth is becoming a hurdle in the way of sustainable development [22]. To achieve the 17 sustainable development goals (SDGs), it is important to deal with environmental problems.

This is the era of globalization, and nations are exchanging goods across borders to feed their populations [23]. Therefore, globalization has increased industrial production in almost every country. If the production is from non-RE sources, it increases the CO_2_ emissions and causes environmental pollution. If the energy resources are altered as a result of foreign direct investment, it can improve air quality by using RE resources [24,25].

**Figure 2:**
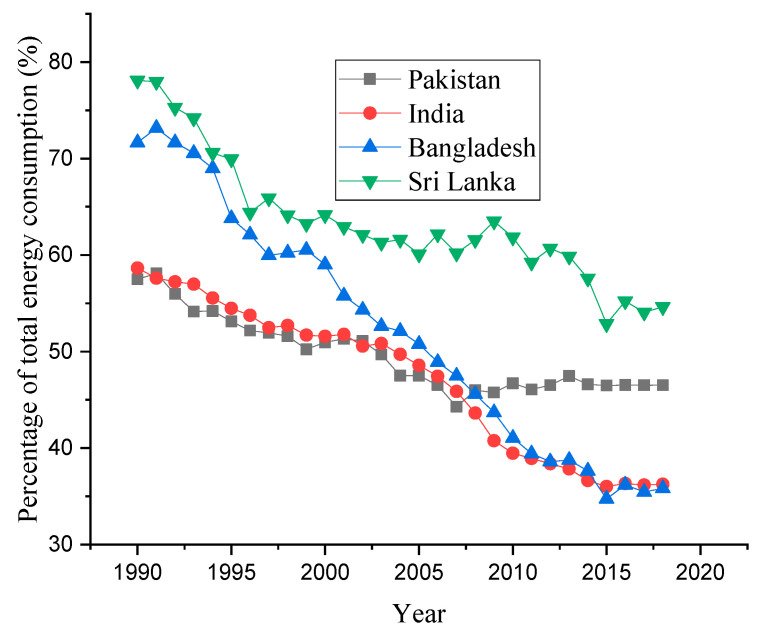
Trends of renewable energy use in South Asian nations (Source: World Bank).

Due to the detrimental role of fossil fuels in the environment, the world is moving towards RE resources [17]. RE can provide clean energy in all economic sectors. It provides more jobs without impacting the environmental quality. RE reduces the imports of fossil fuels and is available domestically in abundance. It is expected that RE production will surpass coal for electricity generation by 2025 [26]. Developing countries are also trying to increase the share of RE in the total energy mix. Figure 2 shows the trend of RE use in developing nations over the estimated period. The RE trend has been continuously decreasing, although currently, these countries are employing efficient technologies for RE production. This study has taken RE as a percentage of total energy consumption. With time, the energy demand has been rising, and these countries continue to focus on non-renewable energy consumption and neglect RE production. Therefore, the ratio of RE as a percentage of total energy is decreasing over time.

This work tries to answer the following questions (1) How does renewable energy impact environmental quality? (2) Is globalization helpful in mitigating the ecological footprints and CO_2_ emissions in South Asian nations? (3) What is the role of agriculture in environmental quality? Hence, this work contributes to the literature on South Asian nations by investigating the impacts of RE use, globalization, and agricultural value-added on CO_2_ emissions and EF in selected South Asian countries.

This work is organized as follows: the background studies are discussed in Section 2. Section 3 presents the methods used and the data description. The results and a discussion are in sound in Section 4, and Section 5 presents the conclusion.

## 2. Background Studies Discussion

Past literature has presented the determining factors of environmental pollution, which can assist in achieving sustainable development. The authors of [27] have documented an indirect association between the GDP and environmental contamination. Apart from economic development, many researchers have associated environmental pollution with globalization, renewable and non-RE resources, and AG.

### 2.1. Renewable Energy and the Environment

Considering the importance of RE use, many researchers have investigated its relationship with ecological contamination. All previous studies presented mixed results regarding the impacts of RE on environmental pollution. Many researchers found a significant positive role of RE in climatic quality, but some found insignificant impacts of RE on the environment. Ridzuan et al. [20] examined the effects of RE and AG on CO_2_ emissions and discovered that RE is lowering CO_2_ emissions in Malaysia. Ellum and Momodu [28] also found that RE is improving the environment in Nigeria. Similarly, the study in [29] found that RE is environmentally friendly in 58 economies. Chen et al. [30] validated the inverted U shape association of RE and CO_2_ emissions. Gill et al. [31] showed the potential of RE in reducing greenhouse gasses in Malaysia. On the other hand, the work in [32] documented that hydropower consumption is not lowering EF in industrial countries. The study in [33] assessed the factors of energy consumption and found that urbanization is increasing energy consumption rapidly. Li et al. [34] researched to determine the impacts of social, economic, and energy structures on CO_2_ emissions. They found that RE is mitigating climatic pollution. Wang et al. [35] found that RE and high oil prices contribute to dissociating economic development from CO_2_ emissions. Li et al. [36] probed the impacts of RE on economic growth and the environment. They found that RE is suitable to increase the GDP while improving environmental quality.

### 2.2. Value-Added Agriculture and the Environment

Value-added agriculture GDP means that the revenue comes from agricultural-related activities. This includes crop production and livestock. This sector is essential to produce food for the growing population. This sector requires huge amounts of energy for irrigation and other related activities. If this energy is coming from non-renewable energy, it will contaminate the climate by emitting GHGs. In this context, recent studies have used different econometric methods to investigate the impacts of AG on environmental quality. Ridzuan et al. [20] examined the effects of RE and AG on CO_2_ emissions and found that RE is lowering CO_2_ emissions in Malaysia. Asumadu-Sarkodie and Owusu [37] applied an autoregressive distributed lag (ARDL) approach to probe the effects of AG on CO_2_ emissions in Ghana. They found that AG had adverse impacts on the climate. On the other hand, the authors of [38] used different panel techniques to show that AG reduced air pollution in 53 nation countries. Jebli and Youseff [39] presented that a 1% increase in AG degrades the environment by 0.36%, but RE use improves air quality. In five North African countries, the research of [40] found that agricultural value improves air quality, but RE contributes to environmental degradation. They argued that the agricultural sector is less polluting than the manufacturing sector. These researchers [11,41] found the inverse association between value-added AG and CO_2_ emissions in Ghana and Spain. Similarly, the authors of [42] found that RE and AG improve air quality by lowering CO_2_ emissions in Malaysia, Indonesia, Thailand, and the Philippines. Another study [43] found that RE improves air quality, but AG degrades air quality in BRICS. For China, the authors of [44] used ARDL, DOLS, and FMOLS and found that AG contributes to more air pollution. By using FMOLS and Maki co-integration tests, the study in [45] found that AG is contaminating the environmental quality. The authors of [14] utilized different econometric techniques and found that different agricultural sectors are contaminating the environment in Pakistan. The study in [16] applied the ARDL approach and found that agricultural activities increase air pollution, but RE consumption reduces air pollution.

Agboola and Bekun [12] applied ARDL and presented that AG is making significant positive impacts on CO_2_ emissions in Nigeria. Balsalobre-Lorente et al. [46] applied FMOLS and DOLS techniques and found that agricultural activities are creating environmental problems. Jebli and Youseff [47] applied ARDL and VECM to show that AG is lowering CO_2_ emissions. Gokmenoglu et al. [13] also found positive impacts of AG in China. Sarkodie et al. [15] utilized quantile ARDL and found that AG and RE are lowering air pollution. For G20, the study in [48] applied FMOLS and VECM to determine that RE improves air quality, but AG degrades it. Aydogan and Vardar [18] presented that RE improves air quality, but AG degrades it. Prastiyo et al. [49] found the negative impacts of AG on CO_2_ emissions in Malaysia. Ridzuan et al. [20] found that fisheries, crops, and RE improve air quality. This work followed the works of [50,51,52], taking the data of the percentage of value-added agriculture of the GDP to capture the impacts of agricultural activities on environmental quality, since agricultural activities put harmful effects on climatic quality. These activities include contaminated water quality due to excessive use of pesticides. Soil pollution is due to land use practices such as overgrazing and farming on land that is not cultivated, ignoring soil preservation methods. High levels of GHGs emissions result due to reckless energy use of fossil fuels [53]. Therefore, it is important to study the harmful impacts of agriculture on ecological footprints.

### 2.3. Globalization and Environment

In this era, when countries are striving for rapid economic growth, the role of globalization has become evident. Due to the trade across the border, countries are exchanging goods with the aid of technological transfer. Different studies have taken two approaches to determine the level of globalization. The globalization index used in the study by [54] provides a comprehensive approach to measuring global ties. This index is measured in three subindexes of political, social, and economic globalization, with an overall index made up of these three indexes. The subsegment of economic globalization comprises trade flows, tariffs, taxes, and trade restrictions. The subsegment of social globalization comprises personal contacts, information flows, cultural globalization, international telephone connections, tourism flows, and migration. The political globalization subsegment is determined by the number of embassies and international non-governmental organizations (NGOs), as well as participation in UN peacekeeping missions. The following are some studies that probe the globalization and environment nexus in different nations.

Ahmed et al. [25] found that globalization helps reduce EF in Japan. Shahbaz et al. [24] conducted an empirical study to show that globalization is environmentally friendly in most high, medium, and low-income countries. The authors of [55] showed that globalization is decreasing CO_2_ emissions in China. Moreover, the study in [56] found that FD and GL negatively affect ecological quality. A recent study by the authors of [57] found that GL and trade are driving environmental pollution in China. The studies in [23,58] found the positive role of globalization in CO_2_ emissions in developing nations and Singapore.

From the above discussion, it can be observed that past literature has investigated the impacts of RE, value-added agriculture, and globalization on environmental quality. Most of the studies have taken CO_2_ emissions as a proxy for environmental degradation, and very little literature has examined the associations of AG, RE, and globalization with EF. Therefore, this work is an attempt to fill this gap by considering the impacts of RE, AG, and globalization on EF in selected South Asian countries.

## 3. Data and Methodology

This research aims to probe the linkages of RE, globalization, AG, and climate. This work takes the selected countries’ annual time series data from 1990 to 2018 for panel study. Therefore, this study has used balanced panel data of selected South Asian nations for panel estimations. Table 1 shows the data description and their sources. Data for RE, CO_2_ emissions, AG, and GL have been obtained from world data indicators and the KOF Economic Institute, and ecological footprint data has been obtained from the Global Footprint Network. This work follows the work of [15,43,59] to estimate two linear equations for RE, globalization, AG, and environmental pollution.

Table 2 shows the descriptive statistics of the study. It can be noted that EF has the maximum value in global hectares, and CO_2_ has the minimum value of 0.13 per capita. CO_2_ has a mean value of 0.72, and GL has a mean value of 49.14.

(1) $$lnCO_{2it}=\beta_{i0\;}+\beta_{1\;}lnRE_{it}+\beta_{2\;}lnGL_{it}+\beta_{3\;}lnAG_{it}+\epsilon_{it}$$

(2) $$lnEF_{2it}=\beta_{i0\;}+\beta_{1\;}lnRE_{it}+\beta_{2\;}lnGL_{it}+\beta_{3\;}lnAG_{it}+\epsilon_{it}$$

Environmental pollution indices receive different impacts from different economic variables. Therefore, two indices for environmental pollution are used in this study. Where CO_2_ is carbon emissions, *RE*, *GL*, and *AG* represent *RE*, globalization, and AG, respectively; *t* is the time, *i* is the cross-section, β is the long run coefficient value, and $\epsilon_{it}$ is the error term. Globalization shows the economic, political, and social dimensions in one index, therefore omitting the problem of biased results of this equation.

Most of the past studies have taken CO_2_ emissions as the proxy for environmental pollution [60], but CO_2_ emissions do not represent soil and water pollution. Therefore, EF provides us with a comprehensive index of environmental pollution [61]. Therefore, for accurate sustainable policy instruments, this study uses EF and CO_2_ emissions as indicators of environmental pollution.

RE is considered environmentally friendly, which reduces the usage of fossil fuels. Therefore, its coefficient values are expected to be negative. Globalization consists of three indices of political, economic, and social globalization [58]. Globalization increases energy consumption with enhanced economic activity. Therefore, it can degrade the environment. At the same time, globalization can bring efficient technologies, which can improve air quality [62].

Apart from the mixed results regarding the impacts of AG on the environment, AG can pollute the environment with its waste [16]. Therefore, its coefficient value is expected to be positive.

### Methodology

Before the application of the long-run evaluation, cross-section dependence is checked in the panel data. In this regard, this research performs the Lagrange multiplier (LM) test used by Breusch and Pagan (1980) and the CD test used by Pesaran and M.H. (2004), and the mathematical form of the CD test is as follows:

(3) $$LM=T\sum_{i=1}^{n-1}\sum_{j=i+1}^{n}\partial_{ij}^{t}$$

(4) $$CD=\sqrt{\frac{2T}{N\left(N-1\right)}}\left(\sum_{i=1}^{n-1}\sum_{j=i+1}^{n}\partial_{ij}^{t}\right)$$

where *T* is the period and *N* represents cross-sections. $\partial_{ij}^{t}$ represents the pairwise linkages of errors between *i* and *j*. This work uses FMOLS and DOLS methods to determine the long-run values. These methods are efficient in providing the coefficient values. FMOLS is a parametric and DOLS is a non-parametric technique, which can deal with the problems of endogeneity and serial correlation in the panel data.

(5) $$\hat{\gamma }{}_{FMOLS}={\left[N^{-1}\sum_{i=1}^{N}{\left(\sum_{t=1}^{t}\cup_{it}-\bar{\cup_{i}}\right)}^{2}\right]}^{-1}\times \left[\left(\sum_{t=1}^{T}\cup_{it}-\bar{\cup_{i}}\right){\hat{S}}_{it}-T{\hat{\Delta }}_{\epsilon \mu }\right]$$

(6) $$\hat{\gamma }{}_{DOLS}=\left[N^{-1}\sum_{i=1}^{N}{\left(\sum_{t=1}^{T}{∁}_{it}{∁}_{it}^{{}^{\prime }}\right)}^{-1}\left(\sum_{t=1}^{T}{∁}_{it}{∁}_{it}^{{}^{\prime }}\right)\right]$$

## 4. Results and Discussion

Before the empirical analysis, it is compulsory to know the order of integration of the data. The data can be integrated at the level of first difference. Upon the validation of the unit root, the next step can be identified for co-integration. Therefore, this work examines the stationarity property of the variables. For this purpose, first generation unit root tests, as used by [63,64], and second generation unit root test of CIPS and CADF are applied. These tests are widely applied and present reliable results. According to Table 3, almost all variables are stationary at the level and first difference.

For a robustness check, this work applies the second-generation unit root tests, and the findings are in Table 4. It is shown that some variables also show a mixed order of integration. This means that RE, CO_2_ emissions, and globalization are stationary at the level and first difference. After determining the order of integration, this study further estimates the cross-sectional dependence (CD) in the panel data. Panel data may have cross sectional dependence due to similar economic policies. If the CD exists in the panel data, then we can use a co-integration test that can consider the CD in the panel data while providing robust findings.

This work performs two LM and CD tests to discover the presence of cross-sectional dependence. Table 5 shows that all variables have cross-sectional dependence at the 1% level. This dependence may be due to similar political and economic policies. After the CD test in the panel data, this work moves forward to discover the co-integration level among the variables. If the co-integration is confirmed, then we can proceed further to find out the long- and short-run coefficient values.

For this purpose, this study performs a co-integration test similar to the one used in [65]. This test is useful for panel co-integration because it considers the cross-sectional dependence among the panel data. According to Table 6, there exists a strong long-run association among the estimated equation.

After confirming the long-run linkages among the variables, the subsequent method is to highlight the short- and long-run associations. Therefore, this work applied two tests of FMOLS and DOLS. FMOLS is a Fourier method and can deal with the delinquent of serial correlation in the panel data. DOLS is an alternative variational method that can also give robust results for the panel data. The findings of these tests are presented below. This research estimates two equations in which two dependent variables, CO_2_ emissions and EF, have been used. Table 7 shows the results when CO_2_ emissions are taken as the dependent variable. RE is negatively associated with CO_2_ emissions at a 1% level. This means that a 1% increase in RE will lower CO_2_ emissions by 10.55% in South Asian countries. RE is mostly generated from solar and hydro powers, which are considered environmentally friendly. Therefore, RE decreases CO_2_ emissions in the long run. This finding is in line with the results of [2,36].

The study found that RE is suitable to mitigate climatic pollution. Moreover, it is also compatible with economic growth. RE mostly comes from wind, solar, and hydropower. These sources do not contaminate the environmental quality. Globalization is contaminating the air quality by increasing CO_2_ emissions by 6.01%. This is because globalization boosts economic activities, which also increases energy consumption. South Asian countries are developing countries, and they rely mainly on fossil fuels for energy utilization. This result also shows that these countries have not succeeded in importing efficient energy resources from developed countries. These efficient resources can be used in the energy sector to replace the traditional means of generating energy. This finding is not similar to the results of [66]. They found that globalization is environmentally friendly because it attracts efficient machinery from developed nations. This outcome shows that these countries need to focus on the importing of efficient means of RE production.

It is worth noting that AG is also a contributing factor to environmental degradation, but this impact is insignificant. This means that agricultural activities are not sustainable in South Asian countries. In South Asian countries, farmers are not well equipped with environmental awareness, and they use traditional means of irrigation. This finding is similar to the results of [17]. They also found that AG is degrading the climate in BRIC nations. Agricultural activities are consuming fossil energy and contributing to environmental degradation.

Table 8 shows the impacts of RE, globalization, and AG on EF in South Asian countries. It can be noted that RE is lowering EF in South Asian countries significantly. RE does not consume biocapacity to provide energy requirements. Globalization is increasing EF; this means that globalization is causing reduced biocapacity because it increases economic activities. These findings are in line with the findings of [48]. Developing countries are still not able to benefit from globalization. Globalization also imports efficient energy technologies, but these countries are lagging behind in this regard. AG is not sustainable in South Asian countries because it is increasing EF in the long run.

Apart from the analysis of FMOLS and DOLS, this work performs analyses using the augmented mean group (AMG) and common corelated effects mean group (CCEMG). These tests are effective in dealing with the problems of CD and heterogeneity in the panel data. The findings are shown in Table 9 and Table 10.

The results show that RE is negatively associated with CO_2_ emissions and EF. Globalization and agricultural activities are contaminating the environment by increasing CO_2_ emissions and ecological footprints in South Asian nations. These results validate the results of FMOLS and DOLS tests and indicate the authenticity of the results as effective policy instruments.

## 5. Conclusions and Recommendations

This work investigates the impacts of RE, globalization, and agriculture on EF and CO_2_ emissions. For quantitative analysis, this study took annual data from 1990 to 2018 for four South Asian countries. These countries are developing countries and rely mainly on AG. These nations are also facing economic transitions due to globalization. For analysis, this work applies first-generation and second-generation unit root tests. Most of the variables were found to have a mixed order of integration. The presence of cross-sectional dependence among the variables further guides us to perform the Westerlund co-integration test for long-run association. After confirming the long-run associations, this work moves forward to conduct FMOLS and DOLS tests to find the long run coefficient values.

The increasing level of CO_2_ emissions and EF is creating environmental problems, which are having detrimental effects on human health. Therefore, it has become important to explore the determining factors of environmental degradation. RE, globalization, and AG are considered important factors that affect the sustainable development of any economy. RE is produced mostly from solar, hydro, and wind sources. These sources provide clean energy, which does not produce environmental pollution.

The AG sector plays a fundamental role in increasing economic growth. The AG sector also consumes energy, and developing countries are lagging behind in providing RE to the AG sector.

Globalization has become an important tool to boost economic activity. It enhances import and export across borders. Globalization increases production in industries, which requires more energy. If the energy comes from fossil fuels, it can also contaminate the environmental quality.

In this context, the findings of this work support the theoretical implications in developing countries. RE is improving air quality by reducing CO_2_ emissions and EF. AG and globalization are creating environmental problems by increasing CO_2_ emissions and EF.

The findings show that RE consumption is lowering CO_2_ emissions and EF in the long run. Globalization is increasing CO_2_ emissions and EF in South Asian countries. Value-added agriculture is also a contributing factor toward environmental degradation, but this link is insignificant.

This study presents some important policy implications for South Asian countries. RE has been proven to be an important alternative for energy consumption. This type of energy does not contaminate air quality. South Asian countries are located such that they receive huge amounts of solar energy. Therefore, these countries need to exploit solar energy to its full capacity. Moreover, these countries need to explore more RE resources to reduce their dependence on non-RE sources.

These countries can make their agricultural sectors sustainable by following efficient farming practices. Environmental awareness should be enhanced among the farmers. Farmers can use animal fertilizers and clean inputs in AG to achieve sustainable agricultural products. Moreover, the inequality for renewable energy use should be eliminated by providing maximum opportunities to the agricultural sectors in these countries. Today, globalization is providing efficient opportunities to nations to import efficient technologies. Therefore, the policymakers must consider their policy instruments regarding external relations. Attention should be given to attracting sustainable technologies for clean energy consumption.

Apart from the contribution of this work, it also has some limitations. This research includes selected South Asian countries because of the availability of data from these areas. Future research should include other developing countries for country-specific analysis. Moreover, future research should be conducted to investigate the impacts of subindices of globalization on EF in developing countries. Moreover, these variables should be used in other blocks of countries to obtain comprehensive results. Another limitation of this study is that the model did not incorporate all the determinants of ecological footprints. Thus, future research should incorporate these determinants.

## Figures and Tables

**Table 1:** Data sources and their description.

Parameters	Symbol	Unit	Source
Ecological footprints	EF	Global hectare	Global Footprint Network
Renewable energy use	RE	% of total energy consumption	World data Indicators
Agriculture	AG	Value-added agriculture percentage of GDP	World data Indicators
Globalization	GL	Overall index (political, social, and economic globalization)	KOF Index

**Table 2:** Descriptive statistics.

	EF	AGR	CO	GL	RE
Mean	3.30 × 10^8^	19.95	0.72	49.14	53.08
Median	1.11 × 10^8^	21.71	0.72	50.52	52.32
Maximum	1.55 × 10^9^	31.67	1.81	61.84	78.08
Minimum	1.47 × 10^7^	7.42	0.13	29.67	34.74
Std. Dev.	4.61 × 10^8^	5.60	0.40	8.74	10.32
Skewness	1.47	−0.53	0.76	−0.32	0.23
Kurtosis	3.68	2.67	3.49	2.04	2.59
Jarque–Bera	44.17	6.00	12.56	6.44	1.91
Probability	0.00	0.04	0.00	0.03	0.38
Sum	3.83 × 10^10^	2314.94	84.26	5700.27	6157.31
Sum Sq. Dev.	2.45 × 10^19^	3607.51	18.42	8799.17	12,267.92
Observations	116	116	116	116	116

**Table 3:** Unit root test.

Variables	LLC		IPS	
	I(0)	I(1)	I(0)	I(1)
$lnCO_{2t}$	−5.27 ***	−6.08 ***	−0.34 *	−4.16 ***
$lnEF_{t}$	2.33	−12.09 ***	0.55	−2.67 **
$lnRE_{t}$	0.94 **	−4.02 ***	0.62 **	−3.67 ***
$lnGL_{t}$	−4.56 ***	−2.56 **	−2.39 **	−0.43
$lnAG_{t}$	−0.15	−12.41 ***	0.51	−5.91 ***

***, **, and * show the significance level of 1%, 5%, and 10%, respectively.

**Table 4:** Unit root tests.

Variables	CADF		CIPS	
	I(0)	I(1)	I(0)	I(1)
$lnCO_{2t}$	−0.78 **	−3.35 **	−1.05 *	−6.04 ***
$lnEF_{t}$	−2.67	−5.25 ***	−1.31	−4.27 ***
$lnRE_{t}$	−3.89 ***	−4.78 ***	−5.62 **	−4.04 ***
$lnGL_{t}$	−4.21 **	−3.35 *	−2.14 *	−3.56 **
$lnAG_{t}$	−0.98	−3.67 **	0.56	−3.65 **

***, **, and * show the significance level of 1%, 5%, and 10%, respectively.

**Table 5:** CD test.

	lnCO_2_	lnEF	lnRE	lnGL	lnAG
LM test	4.61 *** (0.00)	15.66 *** (0.00)	15.80 *** (0.00)	2.31 *** (0.02)	7.94 *** (0.00)
CD test	3.77 *** (0.00)	10.28 *** (0.00)	10.30 *** (0.00)	3.41 *** (0.00)	7.34 *** (0.00)

*** shows the significance level of 1%.

**Table 6:** Westerlund co-integration test.

Stats	Values	Z-Value	Significance	Robust Prob
$G_{t}$	−2.34	5.40	1.00	0.70
$G_{a}$	−3.45 ***	3.44	0.00	0.00
$P_{t}$	−2.76	3.71	1.00	0.75
$P_{a}$	−4.87 ***	4.44	0.00	0.00

*** shows the significance level of 1%.

**Table 7:** FMOLS and DOLS tests (CO_2_).

Variables	FMOLS	Significance	DOLS	Significance
lnRE	−10.55 ***	0.00	−9.82 ***	0.00
lnGL	6.01 ***	0.00	6.58 ***	0.00
lnAG	1.42	0.18	1.51	0.19

*** shows the significance level of 1%.

**Table 8:** FMOLS and DOLS tests (EF).

Variables	FMOLS	Significance	DOLS	Significance
lnRE	−2.08 ***	0.00	−2.07 ***	0.00
lnGL	0.01 *	0.09	0.04 **	0.07
lnAG	0.21	0.58	0.20	0.11

***, **, and * show the significance level of 1%, 5%, and 10%, respectively.

**Table 9:** Robustness check test (CO_2_).

Variables	AMG	CCEMG
$lnRE_{t}$	−7.67 ***	−7.89 ***
$lnGL_{t}$	4.32 ***	3.99 ***
$lnAG_{t}$	1.98 *	1.67 *

***, and * show the significance level of 1%, and 10%, respectively.

**Table 10:** Robustness check test (EF).

Variables	AMG	CCEMG
$lnRE_{t}$	−2.86 ***	−3.90 ***
$lnGL_{t}$	1.78 ***	2.00 ***
$lnAG_{t}$	1.94 *	2.12

***, and * show the significance level of 1%, and 10%, respectively.

## Data Availability

The sources of all data used for the analysis are provided in the main text.
